# Academic burnout syndrome associated with anxiety, stress, depression, and quality of life in Peruvian dentistry students: an analysis using a multivariable regression model

**DOI:** 10.1186/s12909-025-07604-x

**Published:** 2025-07-03

**Authors:** José Menacho-Rivera, Leonor Castro-Ramirez, Enrique Yarasca-Berrocal, José Huamani-Echaccaya, Cinthia Hernández-Vergara, Marysela Ladera-Castañeda, César Cayo-Rojas

**Affiliations:** School of Stomatology, Universidad Privada San Juan Bautista, Lima, Peru

**Keywords:** Psychological burnout, Quality of life, Depression, Anxiety, Stress, Burnout syndrome

## Abstract

**Background:**

Academic burnout syndrome is a condition characterized by chronic physical and emotional exhaustion arising from prolonged exposure to demanding academic pressures. In dental education, factors such as early clinical responsibilities and the necessity for technical precision in practice may significantly exacerbate the syndrome. Therefore, this study aimed to evaluate the association of academic burnout syndrome with anxiety, stress, depression, and quality of life in Peruvian dentistry students, considering potential confounding variables.

**Methods:**

This cross-sectional analytical study, conducted on 566 Peruvian dentistry students, used the Maslach Burnout Inventory (Student-Survey) (MBI-SS) to assess emotional exhaustion, academic inefficacy, and cynicism. The DASS-21 scale was used to diagnose anxiety, stress, and depression, while the EQ-5D test was employed to determine quality of life. The confounding variables considered included age, sex, year of study, marital status, and place of origin. A Poisson regression model with robust variance was used for multivariable analysis, employing adjusted prevalence ratios (APR). Statistical significance was set at *p* < 0.05.

**Results:**

Dentistry students with depression and stress had a 35% (APR = 0.65, 95% CI: 0.54–0.78) and 23% (APR = 0.77, 95% CI: 0.63–0.94) lower probability of experiencing academic inefficacy, respectively. Likewise, students with anxiety had a 23% (APR = 0.77, 95% CI: 0.63–0.95) lower probability of experiencing emotional exhaustion, whereas students with depression and stress had 3.7 times (APR = 3.72, 95% CI: 2.12–6.53) and 59% (APR = 1.59, 95% CI: 1.06–2.40) higher probability of exhibiting cynicism, respectively. Additionally, quality of life did not significantly influence emotional exhaustion, depersonalization, or personal accomplishment. Furthermore, compared to fifth-year students, first-year students had a 54% higher probability of experiencing academic inefficacy (APR = 1.54, 95% CI: 1.23–1.92). In addition, first-year students, along with third- and fourth-year students, had an 80% (APR = 0.20, 95% CI: 1.23–1.92), 56% (APR = 0.44, 95% CI: 0.26–0.75), and 63% (APR = 0.37, 95% CI: 0.20–0.69) lower probability of exhibiting cynicism, respectively. Lastly, female students had a 38% (APR = 0.62, 95% CI: 0.43–0.89) lower probability of exhibiting cynicism than male students.

**Conclusion:**

The findings underscore the multifaceted nature of academic burnout in dental education. While depression and stress were associated with reduced academic inefficacy and cynicism, their adverse clinical implications warrant cautious interpretation. Anxiety was inversely associated with emotional exhaustion, whereas quality of life showed no significant protective role. These results emphasize the necessity of adopting multidimensional strategies to address academic burnout, with interventions tailored to specific academic stages—particularly targeting first-year students—and sex-specific approaches to reduce risk in vulnerable subgroups.

## Background

Freudenberger first described burnout syndrome in 1974 as a response to cumulative stress from adverse working conditions [1, 2]. Later, Maslach characterized the syndrome as a cluster of physical and psychological symptoms, such as negative attitudes toward work, life, and interpersonal relationships, arising from fatigue, exhaustion, hopelessness, and despair [2].

Burnout syndrome, recognized as a clinical occupational condition, refers to a state of emotional exhaustion and depression stemming from prolonged engagement in processes, relationships, or lifestyles that consistently fail to meet expectations. The syndrome comprises three core dimensions: emotional exhaustion, depersonalization, and diminished personal accomplishment [3]. Contributing factors encompass moral distress, overwhelming spiritual and emotional demands, and stressors from both physical and psychological environments [4].

Burnout in academic settings is characterized by emotional exhaustion, academic inefficacy, and cynicism arising from persistent, demanding study requirements [1, 5]. Emotional exhaustion reflects the depletion of students’ emotional and physical reserves; academic inefficacy denotes perceived incapacity to meet academic obligations; and cynicism manifests as detachment or indifference toward one’s studies [1]. Students experiencing these symptoms risk poorer mental and physical health, diminished academic performance, and impaired social functioning, which can jeopardize degree completion and future professional success [6]. Personal and family stressors further exacerbate burnout onset [2, 7].

The Job Demands–Resources (JD-R) model provides a robust framework for understanding burnout among dental students by examining the interplay between academic demands—such as rigorous coursework, clinical responsibilities, and performance pressure—and available resources, including faculty support, adaptive coping strategies, and peer collaboration [8, 9]. When demands exceed resources, emotional exhaustion, and disengagement follow; conversely, adequate resources foster motivation, engagement, and well-being [10].

Globally, burnout affects 25–75% of healthcare professionals, with roughly 30% of nurses and 50% of healthcare assistants—and variable rates among physicians—reporting symptoms across specialties and settings [2, 11]. Evidence indicates that these patterns often emerge during training: a 2019 South Korean study linked heavy academic workload to elevated burnout and depressive symptoms among dental students, along with greater program dissatisfaction and counseling needs [7]. More recently, an Iranian investigation (2022–2023) found that, although only 4.2% of students exhibited high burnout, 23.3% reported overload, and 13.4% perceived underdevelopment—early warning signs of distress that may escalate if unaddressed [12].

Reliable screening tools like the DASS-21 (for anxiety, stress, and depression) and the EQ-5D (for health-related quality of life) are widely adopted in academic research due to their strong psychometric properties [13–17]. As routine mental health screening becomes more common in higher education, health science students—who face intense personal, social, and academic challenges—remain particularly vulnerable to psychological distress, which can substantially impair their well-being and academic outcomes [15, 18, 19].

In Peru, however, critical gaps persist. No studies have examined how the year of study moderates associations between academic burnout and anxiety, stress, depression, or quality of life, nor how sociodemographic factors (e.g., sex, marital status, place of origin) influence these relationships [3, 7]. Moreover, quality of life itself has yet to be explored in relation to academic burnout. This study addresses these gaps by exploring burnout among Peruvian dental students—considering psychological variables, quality of life, and year of study—to generate evidence-based insights for tailored interventions that promote student well-being and mitigate burnout in this unique educational context.

This study is particularly significant because universities must foster an open and objective culture that prioritizes student well-being and facilitates access to professional mental health support when students face difficulties. Furthermore, anxiety, stress, and depression in healthcare students are strongly influenced by the structural organization of their personality [3]. Clinical judgment is critical in guiding therapeutic decision-making—from psychological counseling and psychotherapy to pharmacotherapy and, in severe cases, hospital referral—and universities should strongly encourage affected students to seek such support.

Based on these considerations, the objective of this study was to establish the association between academic burnout syndrome and anxiety, stress, depression, and quality of life in Peruvian dentistry students. The null hypothesis stated that no association exists between academic burnout syndrome and anxiety, stress, depression, or quality of life in this population.

## Methods

### Study design

This analytical and cross-sectional study was written according to the Strengthening the Reporting of Observational Studies in Epidemiology (STROBE) guidelines for observational studies [20]. It was conducted in November and December 2023 at Universidad Privada San Juan Bautista, both at its main campus in Lima, and its branch in Ica, Peru.

### Population and participant selection

**Fig. 1:**
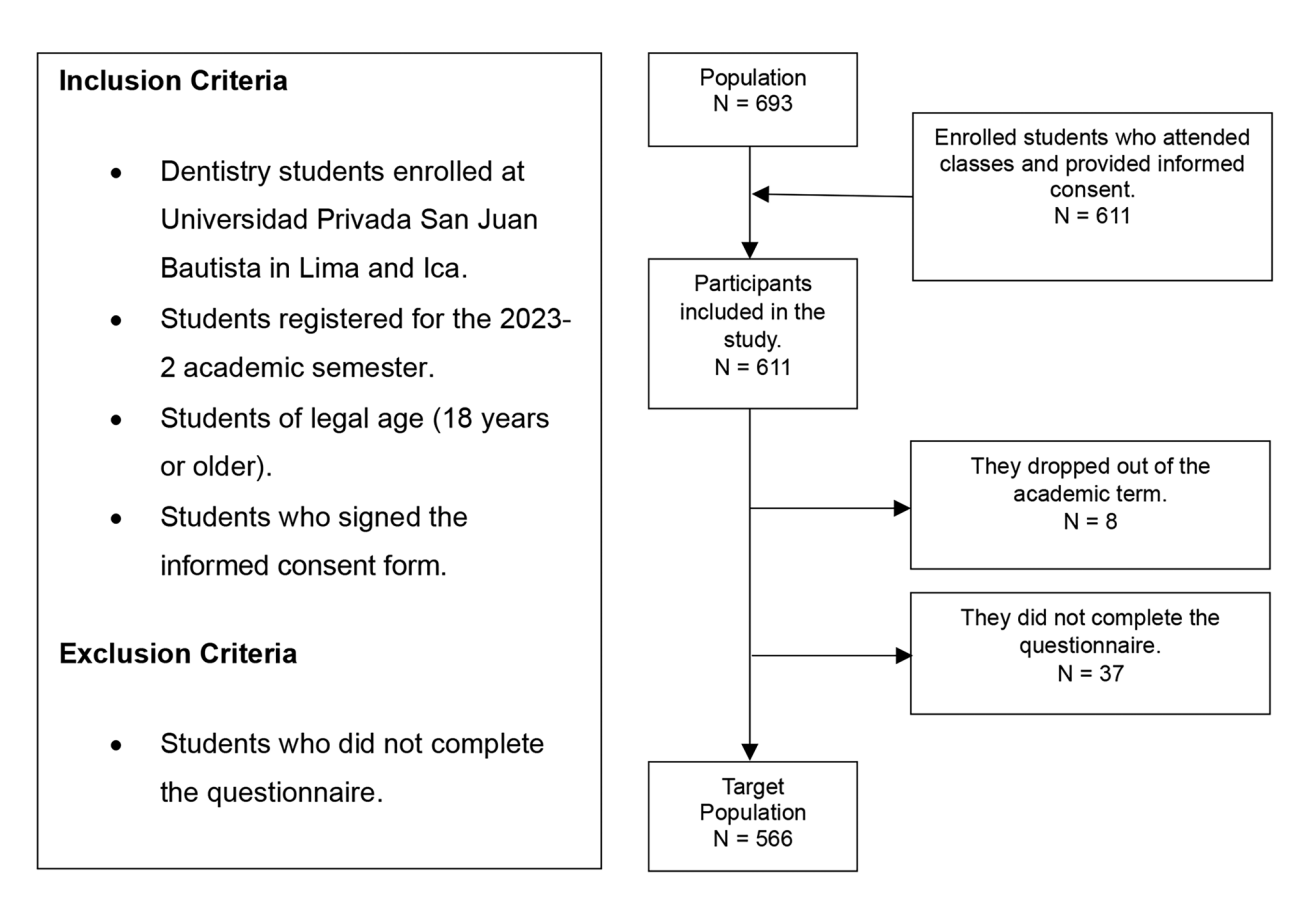
Flowchart of Participant Selection

The total population consisted of 693 dentistry students (374 from Lima and 315 from Ica), distributed as follows: 146 first-year students, 123 s-year students, 184 third-year students, 130 fourth-year students, and 110 fifth-year students. A formula for estimating proportions in a finite population was applied to determine the sample size using the Epidat 4.2 statistical package. The calculation was based on a 95% confidence level, an expected proportion of 50% (to obtain the largest possible sample size), and a margin of error of 5%. The estimated sample size was *n* = 248; however, it was decided to include the entire study population while adhering to the eligibility criteria. Consequently, the final target population was *N* = 566, distributed as follows: 110 first-year students, 101 s-year students, 165 third-year students, 117 fourth-year students, and 73 fifth-year students **[**Figure 1**].**

### Variables

The dependent variable considered in this study was academic burnout syndrome [1, 21], while the independent variables included anxiety, stress, depression [6, 13, 15], and quality of life [16, 17]. Potential confounding variables included age group [1, 7, 13, 15], sex [1, 7, 13, 15], year of study [1, 13, 15], marital status [7, 13, 15], and place of origin [13].

### Diagnostic application instruments based on questionnaires

The Maslach Burnout Inventory (Student-Survey) (MBI-SS), validated for Peruvian university students [1, 21], was used to assess academic burnout symptoms. The questionnaire comprised five items for the emotional exhaustion dimension, six for academic inefficacy, and four for cynicism. The final item values were rated on a Likert scale: 1 (Never), 2 (Rarely), 3 (Sometimes), 4 (Regularly), 5 (Often), 6 (Almost always), and 7 (Always) [1, 16]. The internal consistency for emotional exhaustion, academic inefficacy, and cynicism was optimal, with Cronbach’s alpha (α) values of 0.883 (95% CI: 0.867–0.897), 0.889 (95% CI: 0.874–0.902), and 0.800 (95% CI: 0.772–0.825), respectively. Cut-off points for positive burnout detection were set at ≥ 32 points for emotional exhaustion, ≤ 17 points for academic inefficacy, and ≥ 15 for cynicism. These cut-off values were validated using Livingston’s K^2^ coefficient, yielding 0.886, 0.890, and 0.908, respectively, indicating acceptable values.

To assess symptoms of depression, anxiety, and stress, the DASS-21 self-report scale was utilized. This scale consists of seven items each for anxiety, stress, and depression. Responses were rated on a Likert scale: 0 (Never), 1 (Sometimes), 2 (Frequently), and 3 (Almost always) [6, 13, 15]. The internal consistency for depression, anxiety, and stress was optimal, with Cronbach’s alpha (α) values of 0.880 (95% CI: 0.864–0.895), 0.861 (95% CI: 0.843–0.878), and 0.864 (95% CI: 0.846–0.881), respectively. A score of ≥ 5 indicated depression, ≥ 4 indicated anxiety, and ≥ 8 indicated stress [15]. These cut-off values were validated using Livingston’s K^2^ coefficient, yielding 0.884, 0.886, and 0.864, respectively, confirming their acceptability.

The EQ-5D scale assessed five domains: mobility, self-care, daily activities, pain/discomfort, and anxiety/depression. A student was considered to have a good quality of life if all item scores were 1. If any score differed from 1, the student was classified as having a diminished quality of life [16, 19, 21].

### Procedure

The questionnaire was administered in person. The principal researcher (JMR) conducted the surveys and explained the study’s relevance. The informed consent section on the first page of the questionnaire included the principal researcher’s full name, email, and phone number, as well as contact details for the Institutional Research Ethics Committee. Students who provided consent proceeded to the next page, which contained instructions for completing the questionnaires. They had full autonomy to decline participation or refrain from completing any questionnaire. Only the principal researcher had access to the participant’s data, which was securely stored on a password-protected portable digital device to ensure confidentiality. Each student was allowed a single participation. Participants were asked to provide their initials and age (e.g., JMR22) to filter duplicate responses in cases where a student was enrolled in multiple academic cycles. No incentives were offered for participation, and students who requested their results received them via email from the principal researcher.

### Data analysis

The statistical software SPSS v.28.0 was used for data analysis, with data imported from a Microsoft Excel 2019 spreadsheet. Descriptive analysis for qualitative variables included absolute and relative frequencies, while the quantitative variable (age) was analyzed using mean, median, and standard deviation. A Poisson multiple regression model with robust variance was applied using the adjusted prevalence ratio (APR) for multivariable analysis to assess the effect of depression, anxiety, stress, and quality of life on positive burnout symptoms, including emotional exhaustion, academic inefficacy, and cynicism while considering potential confounding variables. Statistical significance was set at *p* < 0.05.

### Bioethical considerations

The Institutional Research Ethics Committee of Universidad Privada San Juan Bautista approved the execution of this study under letter No. 1434-2023-CIEI-UPSJB dated 23 October 2023. This study adhered to the bioethical principles of the Declaration of Helsinki, including respect, autonomy, non-maleficence, and confidentiality. Additionally, voluntary written informed consent was obtained on the first page of the questionnaire.

## Results

The average age of the participants was 22.3 ± 4.7 years, with 52.7% being 21 years old or younger and 63.3% of the total sample being female. Additionally, the highest percentage of students were in their third year of studies (29.2%), and the vast majority were single (87.1%). Lastly, 50.4% of the respondents were from provinces **[**Table 1**].**

**Table 1:** Sociodemographic characteristics of dentistry students

Variable	Categories	Frequency	Percentage
**Age group**	≤ 21 years	298	52.7
	> 21 years	268	47.3
**Sex**	Female	358	63.3
	Male	208	36.7
**Year of study**	1st year	110	19.4
	2nd year	101	17.8
	3rd year	165	29.2
	4th year	117	20.7
	5th year	73	12.9
**Marital status**	Single	493	87.1
	Married or cohabiting	73	12.9
**Place of origin**	Capital	281	49.6
	Province	285	50.4
**Age**	**Mean**	**Median**	**SD**
	22.3	21.0	4.7

SD: Standard Deviation

**Fig. 2:**
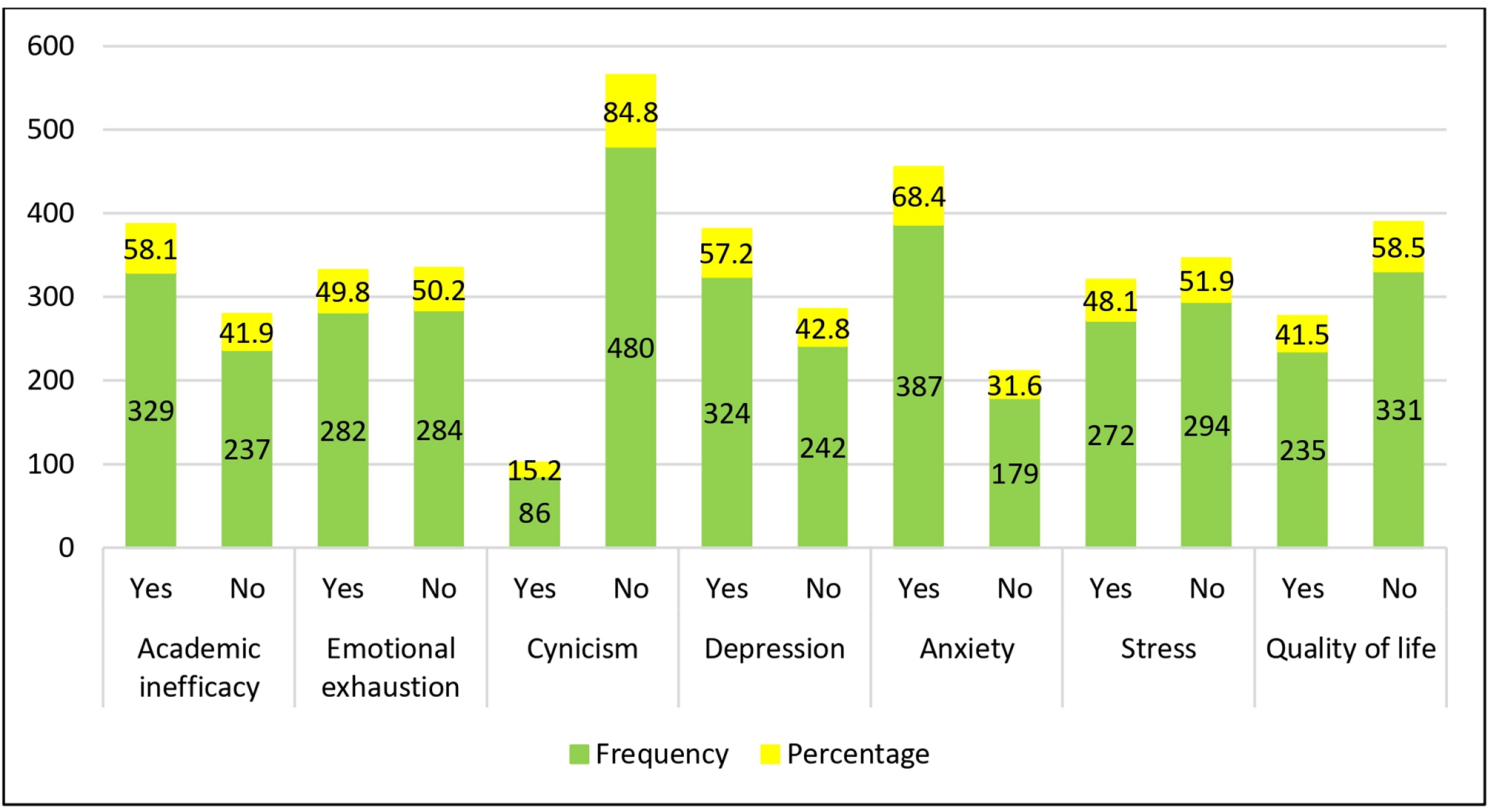
Frequency of depression, anxiety, stress, quality of life, and academic burnout syndrome according to academic inefficacy, emotional exhaustion, and cynicism

Among the total respondents, 58.1% (95% CI: 54.1 − 62.2%), 49.8% (95% CI: 45.7 − 53.9%), and 15.2% (95% CI: 12.2– 18.2%) exhibited academic inefficacy, emotional exhaustion, and cynicism, respectively. Additionally, 57.2% (95% CI: 53.2 − 61.3%), 68.4% (95% CI: 64.5 − 72.2%), and 48.1% (95% CI: 43.9 − 52.2%) of the total students experienced depression, anxiety, and stress, respectively. Finally, 41.5% (95% CI: 37.5 − 45.6%) of the participants reported having a good quality of life **[**Figure 2**].**

In the unadjusted model of the Poisson regression analysis with robust variance using Prevalence Ratios (PR), positive burnout was defined as academic inefficacy (Yes [≤ 17 points] = 1 / No [> 17 points] = 0), emotional exhaustion (Yes [> 31 points] = 1 / No [≤ 31 points] = 0), and cynicism (Yes [> 14 points] = 1 / No [≤ 14 points] = 0), which served as the dependent variables. Depression (Yes [≥ 5 points] = 1 / No [< 5 points] = 0), anxiety (Yes [≥ 4 points] = 1 / No [< 4 points] = 0), stress (Yes [≥ 8 points] = 1 / No [< 8 points] = 0), and quality of life (Yes = 1 / No = 0) were the independent variables. In contrast, potential confounding variables included age group, sex, year of study, marital status, and place of origin **[**Tables 2 and 3, and 4**].**

After adjusting the prevalence ratio (APR) in the regression model, it was found that dentistry students with depression and stress had a 35% (APR = 0.65, 95% CI: 0.54–0.78) and 23% (APR = 0.77, 95% CI: 0.63–0.94) lower probability of experiencing academic inefficacy compared to those without depression and stress, respectively. Additionally, first-year students showed a 54% higher probability of academic inefficacy (APR = 1.54, 95% CI: 1.23–1.92) than fifth-year students [Table 2].

**Table 2:** Poisson regression analysis of academic inefficacy associated with depression, anxiety, stress, quality of life, and sociodemographic variables in dentistry students

Variable	Categories		Academic Inefficacy										
			Crude model						Adjusted model				
		VIF	β	PR	95% CI		p*		β	APR	95% CI		p**
					**LL**	**UL**					**LL**	**UL**	
**Depression**	Yes	1.77	-0.67	0.51	0.44	0.59	< 0.001		-0.43	0.65	0.54	0.78	< 0.001
	No			*Ref.*						*Ref.*			
**Anxiety**	Yes	1.64	-0.48	0.62	0.54	0.70	< 0.001		-0.08	0.93	0.80	1.07	0.311
	No			*Ref.*						*Ref.*			
**Stress**	Yes	1.65	-0.61	0.54	0.46	0.64	< 0.001		-0.26	0.77	0.63	0.94	0.009
	No			*Ref.*						*Ref.*			
**Quality of Life**	Yes	1.31	0.36	1.43	1.25	1.64	< 0.001		0.08	1.08	0.94	1.25	0.270
	No			*Ref.*						*Ref.*			
**Age group**	≤ 21 years	1.41	-0.16	0.85	0.74	0.98	0.024		-0.14	0.87	0.75	1.01	0.063
	> 21 years			*Ref.*						*Ref.*			
**Sex**	Female	1.03	-0.09	0.91	0.79	1.05	0.203						
	Male			*Ref.*									
**Year of Study**	1st year	2.43	0.23	1.26	1.03	1.53	0.026		0.43	1.54	1.23	1.92	< 0.001
	2nd year	2.29	-0.28	0.75	0.58	0.99	0.041		-0.08	0.92	0.71	1.20	0.543
	3rd year	2.55	-0.23	0.80	0.63	1.01	0.057		0.02	1.02	0.81	1.28	0.879
	4th year	2.15	-0.13	0.88	0.69	1.12	0.302		0.05	1.05	0.84	1.33	0.658
	5th year			*Ref.*						*Ref.*			
**Marital Status**	Single	1.12	-0.04	0.96	0.78	1.17	0.684						
	Married or cohabiting			*Ref.*									
**Place of Origin**	Capital	1.04	0.02	1.02	0.89	1.17	0.777						
	Province			*Ref.*									

*Simple regression model (crude); variables with p < 0.2 were included in the adjusted model. **Multiple regression model adjusted (**p < 0.05,* significant association). APR: Adjusted Prevalence Ratio under the Poisson regression model with robust variance. VIF: Variance Inflation Factor (Mean VIF = 1.70); β: Determination coefficient. 95% CI: 95% Confidence Interval; LL: Lower Limit*,* UL: Upper Limit*

After adjusting the prevalence ratio in the regression model, it was observed that dentistry students with anxiety had a 23% lower probability (APR = 0.77, 95% CI: 0.63–0.95) of experiencing emotional exhaustion compared to those without anxiety [Table 3].

**Table 3:** Regression analysis of emotional exhaustion associated with depression, anxiety, stress, quality of life, and sociodemographic variables among dentistry students

Variable	Categories		Emotional exhaustion										
			Crude model						Adjusted model				
		VIF	β	PR	95% CI		p*		β	APR	95% CI		p**
					**LL**	**UL**					**LI**	**LL**	
**Depression**	Yes	1.77	-0.42	0.66	0.56	0.77	< 0.001		-0.20	0.82	0.65	1.02	0.075
	No			*Ref.*						*Ref.*			
**Anxiety**	Yes	1.64	-0.44	0.64	0.55	0.75	< 0.001		-0.26	0.77	0.63	0.95	0.015
	No			*Ref.*						*Ref.*			
**Stress**	Yes	1.65	-0.32	0.72	0.61	0.86	< 0.001		0.01	1.01	0.81	1.26	0.922
	No			*Ref.*						*Ref.*			
**Quality of Life**	Yes	1.31	0.31	1.37	1.16	1.61	< 0.001		0.14	1.15	0.95	1.38	0.146
	No			*Ref.*						*Ref.*			
**Age group**	≤ 21 years	1.41	-0.18	0.84	0.71	0.99	0.036		-0.16	0.85	0.71	1.02	0.083
	> 21 years			*Ref.*						*Ref.*			
**Sex**	Female	1.03	-0.04	0.96	0.81	1.14	0.678						
	Male			*Ref.*									
**Year of Study**	1st year	2.43	0.00	1.00	0.77	1.28	0.972		0.18	1.20	0.92	1.57	0.181
	2nd year	2.29	-0.19	0.83	0.62	1.10	0.188		-0.04	0.96	0.71	1.29	0.783
	3rd year	2.55	-0.25	0.78	0.60	1.01	0.060		-0.08	0.92	0.70	1.20	0.549
	4th year	2.15	-0.20	0.82	0.62	1.08	0.150		-0.09	0.91	0.69	1.21	0.524
	5th year			*Ref.*						*Ref.*			
**Marital Status**	Single	1.12	0.04	1.04	0.81	1.35	0.735						
	Married or cohabiting			*Ref.*									
**Place of Origin**	Capital	1.04	0.00	1.00	0.85	1.18	1.000						
	Province			*Ref.*									

*Simple regression model (crude); variables with *p* < 0.2 were included in the adjusted model. **Multiple regression model adjusted (**p < 0.05,* significant association). APR: Adjusted Prevalence Ratio under the Poisson regression model with robust variance. VIF: Variance Inflation Factor (Mean VIF = 1.70). APR; β: Determination coefficient. 95% CI: 95% Confidence Interval; LL: Lower Limit*,* UL: Upper Limit.*

When adjusting the prevalence ratio in the regression model, it was observed that dental students with depression and stress had 3.7 times (APR = 3.72, 95% CI: 2.12–6.53) and 59% (APR = 1.59, 95% CI: 1.06–2.40) higher probability of exhibiting cynicism, respectively, compared to those without depression and stress. On the other hand, female students had a 38% (APR = 0.62, 95% CI: 0.43–0.89) lower probability of presenting cynicism than males. Additionally, students in the first, third, and fourth years showed an 80% (APR = 0.20, 95% CI: 1.23–1.92), 56% (APR = 0.44, 95% CI: 0.26–0.75), and 63% (APR = 0.37, 95% CI: 0.20–0.69) lower probability of exhibiting cynicism, respectively, compared to fifth-year students [Table 4].

**Table 4:** Regression analysis of cynicism associated with depression, anxiety, stress, quality of life, and sociodemographic variables among dentistry students

Variable	Categories		Cynicism										
			Crude model						Adjusted model				
		VIF	β	PR	95% CI		p*		β	APR	95% CI		p**
					**LL**	**UL**					**LI**	**LL**	
**Depression**	Yes	1.77	1.35	3.84	2.22	6.64	< 0.001		1.31	3.72	2.12	6.53	< 0.001
	No			*Ref.*						*Ref.*			
**Anxiety**	Yes	1.64	0.63	1.88	1.14	3.10	0.014		-0.18	0.84	0.49	1.42	0.507
	No			*Ref.*						*Ref.*			
**Stress**	Yes	1.65	0.86	2.36	1.54	3.61	< 0.001		0.47	1.59	1.06	2.40	0.025
	No			*Ref.*						*Ref.*			
**Quality of Life**	Yes	1.31	-0.18	0.83	0.56	1.25	0.381						
	No			*Ref.*									
**Age group**	≤ 21 years	1.41	0.13	1.14	0.77	1.68	0.524						
	> 21 years			*Ref.*									
**Sex**	Female	1.03	-0.31	0.73	0.50	1.08	0.120		-0.48	0.62	0.43	0.89	0.010
	Male			*Ref.*						*Ref.*			
**Year of Study**	1st year	2.43	-1.30	0.27	0.12	0.63	0.002		-1.59	0.20	0.09	0.47	< 0.001
	2nd year	2.29	-0.07	0.94	0.54	1.63	0.814		-0.36	0.70	0.40	1.20	0.191
	3rd year	2.55	-0.43	0.65	0.37	1.13	0.126		-0.81	0.44	0.26	0.75	0.003
	4th year	2.15	-0.60	0.55	0.29	1.03	0.063		-0.99	0.37	0.20	0.69	0.002
	5th year			*Ref.*						*Ref.*			
**Marital Status**	Single	1.12	-0.27	0.76	0.45	1.28	0.302						
	Married or cohabiting			*Ref.*									
**Place of Origin**	Capital	1.04	0.11	1.11	0.75	1.64	0.590						
	Province			*Ref.*									

*Simple regression model (crude); variables with p < 0.2 were included in the adjusted model. **Multiple regression model adjusted (**p < 0.05,* significant association). APR: Adjusted Prevalence Ratio under the Poisson regression model with robust variance. VIF: Variance Inflation Factor (Mean VIF = 1.70). APR; β: Determination coefficient. 95% CI: 95% Confidence Interval; LL: Lower Limit*,* UL: Upper Limit.*

## Discussion

Several studies have indicated that burnout syndrome negatively affects university students and may be influenced by behavioral disorders [1, 11, 23–25]. Therefore, this study aimed to evaluate the association between academic burnout syndrome and anxiety, stress, depression, and quality of life in Peruvian dental students. The findings led to the rejection of the null hypothesis.

This study identified anxiety as the most prevalent psychological condition among dental students, followed by depression and stress. These findings are consistent with trends reported by Castro et al. [12] and Fauzi et al. [13]. In contrast, Ochnik et al. [23] observed higher stress prevalence, with depression and anxiety ranking lower—a divergence potentially influenced by their focus on international students with diverse cultural backgrounds and varying socioeconomic contexts. Additionally, methodological differences, such as the use of distinct assessment tools (e.g., Generalized Anxiety Disorder Scale [GAD-7], Patient Health Questionnaire [PHQ-8], and Perceived Stress Scale [PSS-10]), may contribute to these discrepancies. Notably, variability in cutoff thresholds across anxiety screening scales can affect diagnostic sensitivity and specificity, complicating cross-study comparisons [24].

Among the three dimensions of academic burnout syndrome, academic inefficacy was most prevalent, followed by emotional exhaustion and cynicism—findings that mirror those of Bhattacharyya et al. [25] and Jiménez et al. [26], albeit at lower levels, likely because their samples consisted of second-year students who had not yet begun patient care, whose primary concerns were limited free time and exam pressure, and who may have bolstered their motivation through extracurricular participation [25, 26]. Conversely, Al-Alawi et al. [1] reported higher cynicism among Omani medical students living away from family, suggesting that lack of familial support can intensify academic burnout. Interestingly, despite these burnout dimensions, 41.5% of students reported a good quality of life—contrasting sharply with the lower proportions documented by Milošević et al. [27] and Gadelha et al. [28] during the COVID-19 pandemic, when elevated overload, fear, and psychological distress were linked to emotional exhaustion, feelings of helplessness, depersonalization, negative attitudes toward work and life, and diminished personal achievement [29–31]. Considering that the pandemic context may have served as a confounding factor, intensifying psychological distress independently of academic burnout is important. Consequently, the strong associations observed during that period may reflect the compounded effects of pandemic-related uncertainty and social isolation rather than academic stressors alone.

It was also observed that dental students with depression and stress were 35% and 23% less likely, respectively, to experience academic inefficacy. Although stress and depression can undermine confidence in one’s academic abilities, they may also prompt students to set realistic goals, enhance resilience, and maintain motivation [32, 33]. Some cope with depressive symptoms by staying connected with peers and immersing themselves in coursework, which can further support a sense of efficacy [34]. Additionally, first-year students were 54% more likely to report academic inefficacy than their fifth-year peers. This disparity likely reflects seniors’ advanced metacognitive awareness and self-regulatory skills. Fifth-year students understand curricular demands more precisely, organize and prioritize tasks more effectively, and appraise their strengths and limitations accurately. Having overcome multiple clinical and theoretical challenges throughout their training, they exhibit robust self-confidence and a positive self-perception, enabling them to view academic obstacles as opportunities for growth rather than stressors [35, 36]. As students progress, they naturally develop the experience and resilience needed to manage increasing academic demands [37]. Moreover, recent longitudinal work has identified distinct burnout trajectories among health-profession students and shown that resilience is a dynamic protective factor against escalating symptoms [38]. Interventions such as mindfulness-based stress reduction and structured peer-support programs have effectively bolstered resilience and reduced burnout during clinical training [39]. Such evidence suggests that integrating formal resilience-building curricula into dental education could help preempt the progression of academic burnout.

In contrast, de Afshar et al. (2025) found that sixth-year students were more likely to experience academic inefficacy than fourth-year students. This discrepancy may stem from differences in socioeconomic background, curriculum design, institutional support, and curricular intensity across programs [12]. Furthermore, students with anxiety were 23% less likely to suffer emotional exhaustion, perhaps because adaptive optimism associated with anxiety enhances resilience and helps preserve emotional balance [40]. However, because the study uses a cross-sectional design, it may reflect reverse causality, so researchers should not interpret this association as a protective effect without additional longitudinal evidence.

Dental students with depression and stress were 3.7 times and 59% more likely, respectively, to exhibit cynicism, a finding that may reflect the established link between cynical hostility and stressful events [41, 42]. The association with depression is particularly unexpected, as earlier studies have primarily linked depressive symptoms with emotional exhaustion rather than with other burnout dimensions [43]. Female students were 38% less likely to experience cynicism, which may be influenced by societal gender roles that shape coping behaviors—men tending toward emotional detachment and women more inclined to seek familial support and maintain emotional involvement [44, 45]. Moreover, first-, third-, and fourth-year students were less prone to cynicism than their fifth-year peers, suggesting that cynicism may function as an adaptive mechanism in response to the increased academic stress, fatigue, and responsibilities of senior students [46, 47]. In this study, 58% of participants scored highest on academic inefficacy—the initial burnout phase—thereby highlighting the urgent need for early-stage intervention strategies [48, 49]. In spite of the frequent co-occurrence of mental health disorders with somatic symptoms such as pain, headaches, and fatigue—symptoms among the strongest predictors of poor quality of life in healthcare undergraduates [50–53]—no significant association was observed between any burnout dimension and quality of life, and students identified as emotionally exhausted by the Maslach Burnout Inventory did not uniformly display behavioral disturbances detectable by the EQ-5D instrument [16, 19]. This apparent disconnection may stem from the limited sensitivity of generic quality of life measures like the EQ-5D in capturing the nuanced psychological and academic stressors specific to dental education. Consequently, students may report acceptable overall quality of life while simultaneously experiencing high levels of burnout driven by academic demands, performance pressure, and clinical responsibilities—factors not fully reflected in broad health-related quality of life assessments.

One of the strengths of this study’s design is the use of the Maslach Burnout Inventory (Student-Survey), which has been adapted and validated for Peruvian university students in health sciences [21]. This enhances its applicability, as the questionnaire was initially developed for a Euro-American population [1]. The study also used the DASS-21 scale, a widely recognized and reliable tool for detecting depression, stress, and anxiety, tested in 42 languages [54, 55]. The researchers also used the EQ-5D questionnaire to assess quality of life, a tool validated across various populations worldwide [16, 19]. Another strength of the study is the use of multivariable regression analysis, which identifies potential factors influencing the development of burnout syndrome while accounting for possible confounding variables. This approach is crucial, as bivariate associations do not necessarily imply causation or influence [56, 57].

This study has several limitations. The sample is not representative of all dental students in Peru; however, it provides a valuable basis for future research at local, regional, national, and international levels. The cross-sectional design also limits the ability to assess the long-term effects of anxiety, stress, depression, and reduced quality of life on emotional exhaustion, academic inefficacy, and cynicism. In-person data collection by the principal investigator may have introduced social desirability bias due to the sensitive nature of the psychological assessments. The investigator’s presence could have influenced response accuracy and reduced participants’ sense of anonymity.

Furthermore, some findings—such as the inverse associations between depression, stress, and academic inefficacy, as well as between anxiety and emotional exhaustion—were unexpected and diverged from existing literature. Measurement bias may explain these discrepancies, as the instruments used may not have accurately reflected the actual psychological symptoms or academic distress levels, potentially distorting the associations. Therefore, we recommend that future research investigate the diagnostic accuracy of academic burnout syndrome to adjust the dichotomization of levels within its dimensions. Unmeasured or insufficiently controlled variables may also introduce residual confounding, influencing independent and dependent outcomes. As a result, researchers should interpret these findings with caution.

This research is important as it explores the relationship between academic burnout and anxiety, stress, depression, and quality of life among Peruvian dental students. The findings may encourage universities to foster a culture of openness and objectivity that promotes student well-being and facilitates access to professional support for those facing challenges [58]. Preventive strategies should include the development of psychological support plans starting from the first year of dental education to mitigate the impact of mental health concerns. The DASS-21 scale can serve as an early screening tool for psychological distress. If results are positive, the adapted Maslach Burnout Inventory can be used in academic settings to ensure timely professional support for students at risk of burnout.

## Conclusion

The findings underscore the multifaceted nature of academic burnout in dental education. While depression and stress were associated with reduced academic inefficacy and cynicism, their adverse clinical implications warrant cautious interpretation. Anxiety was inversely associated with emotional exhaustion, whereas quality of life showed no significant protective role. These results emphasize the necessity of adopting multidimensional strategies to address academic burnout, with interventions tailored to specific academic stages—particularly targeting first-year students—and sex-specific approaches to reduce risk in vulnerable subgroups.

## Data Availability

All data analyzed during this study are available from the corresponding author on reasonable request (cesarcayorojas@gmail.com).

## Abbreviations

APR: Adjusted Prevalence Ratio
CI: Confidence interval
COVID-19: COronavirus DIsease 2019
DASS: Depression, Anxiety, and Stress Scale
HRQoL: health-related quality of life
MBI-SS: Maslach Burnout Inventory (Student-Survey)
STROBE: STrengthening the Reporting of OBservational studies in Epidemiology
SPSS: Statistical Package for the Social Sciences
